# Residential road traffic noise exposure and colorectal cancer survival – A Danish cohort study

**DOI:** 10.1371/journal.pone.0187161

**Published:** 2017-10-30

**Authors:** Nina Roswall, Pernille Envold Bidstrup, Ole Raaschou-Nielsen, Steen Solvang Jensen, Kim Overvad, Jytte Halkjær, Mette Sørensen

**Affiliations:** 1 Danish Cancer Society Research Center, Copenhagen, Denmark; 2 Department of Environmental Science, Aarhus University, Roskilde, Denmark; 3 Department of Public Health, Section for Epidemiology, Aarhus University, Aarhus, Denmark; Universita degli Studi di Salerno, ITALY

## Abstract

**Background:**

Residential traffic noise exposure may entail sleep disruption and compromised circadian functioning; two factors which have been associated with a poor colorectal cancer (CRC) prognosis. Hence, the aim of the present study was to investigate the association between residential road traffic noise and CRC survival.

**Methods and materials:**

Road traffic noise was calculated for all residential addresses from 1987 to February 2012 for incident CRC cases (n = 1,234) in a cohort of 57,053 Danes. We used Cox Proportional Hazard Models to investigate the association between residential road traffic noise at different time-windows, and overall and CRC-specific mortality. Furthermore, we investigated interaction with sex, age, prognostic factors, and comorbidity. Mortality Rate Ratios (MRR) were calculated in unadjusted models, and adjusted for railway noise, lifestyle factors, and socioeconomic variables.

**Results:**

During a median follow-up of 4 years, 594 patients died; 447 from CRC. We found no association between road traffic noise exposure and overall (MRR 1.00 (0.88–1.13) per 10 dB) or CRC-specific mortality (MRR 0.98 (0.85–1.13) per 10 dB) over the entire follow-up period, or 1 year preceding death. Results did not differ when examining colon and rectal cancer separately. Interaction analyses suggested that patients with less clinically advanced disease could be more susceptible to harmful effects of traffic noise.

**Conclusion:**

The present study suggests no overall association between residential road traffic noise and concurrent mortality in CRC patients. As it is the first study of its kind, with relatively limited power, further studies are warranted.

## Background

Colorectal cancer is the third most common cancer worldwide, and the fourth leading cause of cancer mortality [1]. Survival vary according to stage at diagnosis, but averages 50–59% 5 years after diagnosis in most countries [2]. This highlights the need for investigation of modifiable factors which could affect colorectal cancer progression and prognosis.

Noise exposure has been classified as the second most important environmental pollutant in Europe after air pollution [3], and people with already existing illnesses have been proposed to be especially vulnerable to the health-detrimental consequences of noise [4–6]. One of the pathways through which traffic noise affects health is via sleep disturbance [4, 5, 7]. Cancer patients often report sleeping problems [8], and a study examining the role of sleep before and during chemotherapy on colorectal cancer prognosis found that sleep problems were unfavorably associated with disease progression and prognosis [9]. Furthermore, sleep disruption may disturb the circadian rhythm and result in suppression of melatonin, which is known to possess anti-carcinogenic properties [10–12], and has been found to reduce the growth rate of already established tumors [13], and block cell invasion and metastasis [14].

Studies investigating clock gene expression profiles in colorectal cancer patients have generally found compromised expression patterns in neoplastic, compared to adjacent non-neoplastic tissues [15–18]. Some studies have also shown an association between compromised circadian functioning and clinicopathological features, including TNM-stage [15, 18], lymph node involvement [15], microsatellite instability [15], liver metastases [16], as well as poorer survival rates [15, 16, 19–22]. These findings imply an association between circadian dysregulation and colorectal cancer aggressiveness and prognosis, which could be causal. Finally, clock genes have also been found related to the development of resistance to chemotherapy, suggesting that this could be another pathway through which traffic noise affects survival after colorectal cancer [23]. Taken together, these studies suggest a potential association between traffic noise exposure and colorectal cancer prognosis, which has yet to be investigated.

The objective of the present study was to investigate the association between residential traffic noise exposure and overall and colorectal cancer-specific mortality, and to explore effect modification by age, sex, prognostic factors, and comorbidity.

## Methods and material

### Study population

The study is based on the prospective Diet, Cancer and Health (DCH) cohort, which has been described in detail previously [24]. Briefly, 160,725 Danes were invited to participate from 1993–97. Inclusion criteria were 50–64 years of age, residence in the greater Copenhagen or Aarhus area, and no previous cancer diagnosis in the Danish Cancer Registry; 57,053 participants (29,875 women) accepted the invitation and were included into the study, representing 7% of the Danish population in this age group.

The DCH-participants have been followed up in Danish registries on cancer and mortality since baseline, and the present study is based on all participants who was registered with a colorectal cancer as their first, primary cancer in the Danish Cancer Registry [25], between baseline and February 10^th^, 2012.

### Compliance with ethical standards

The study complies with the current laws of Denmark, in which the study was performed. All procedures performed in studies involving human participants were in accordance with the ethical standards of the institutional and/or national research committee and with the 1964 Helsinki declaration and its later amendments or comparable ethical standards. Written informed consent was obtained from all individual participants included in the study.

The study was approved by the local ethical committees of Copenhagen and Frederiksberg Municipalities Municipalities (in Danish: "*Den Videnskabsetiske komite for Københavns og Frederiksberg Kommuner*") Approval no.: (KF) 01-345/93.

### Exposure assessment

The assessment of traffic noise exposure for the present cohort has been described in details elsewhere [26]. Briefly, residential address history was collected for all cohort members between July 1^st^, 1987 and February 10^th^, 2012, using the Danish civil registration system [27]. Road traffic noise exposure was calculated using SoundPLAN, which implements the joint Nordic prediction method for road traffic noise [28]. By use of this method, equivalent A-weighted noise levels can be calculated for each address in a position on the most exposed facade of the building, and at the following periods: day (07–19), evening (19–22), and night (22–07), when a series of traffic and topographic parameters are known. These input variables included: points for noise estimation (geographical coordinate and height (floor) for each residential address), road links (information on annual average daily traffic, vehicle distribution (light/heavy), travel speed, and road type), and building polygons for all Danish buildings provided by the Danish Geodata Agency. We obtained traffic counts for all roads with more than 1,000 vehicles per day from a national road and traffic database [29]. No information was available on noise barriers or road surfaces. Noise was expressed as L_den_ (den = day, evening, night), with use of a 5 and 10 dB penalty to evening and night levels, respectively,

Railway traffic noise exposure was calculated for all present and historical addresses using SoundPLAN, implementing a Nordic calculation method for predicting noise propagation for railway traffic noise (NORD2000). The input variables for the noise model were: point for noise estimation (geographical coordinate and height), railway links (information on annual average daily train lengths, train types, travel speed) and building polygons for all Danish buildings. All noise barriers along the railway are included in the model. Railway traffic noise was expressed as L_den_ at the most exposed facade of the dwelling.

For the assessment of road traffic noise the terrain was assumed flat, a reasonable assumption in Denmark. Urban areas, roads, and areas with water were assumed to be hard surfaces, whereas all other areas were assumed acoustically porous.

### Outcomes

We investigated two outcomes: Overall mortality and colorectal cancer-specific mortality. Information regarding vital status was collected by linkage to the Danish Civil Registration System [27], and cause of death was determined by the Cause of Death Registry [30]. Colorectal cancer-specific mortality was defined as having an underlying cause of death defined by an ICD-10 code of C18, C19 or C20 in the Cause of Death Registry.

### Covariates

The selection of covariates was done based on a review of existing literature, biological plausibility, and availability of data.

At baseline of the Diet, Cancer and Health study, all participants filled in a food frequency and a lifestyle questionnaire, and anthropometric measures were collected by trained personnel. The data on diet and lifestyle factors hail from this questionnaire [24].

Furthermore, information on prognostic factors (tumor stage and lymph node involvement) was available from time of diagnosis, through the Danish Colorectal Cancer Group. This is a national, clinical registry which includes information on all colorectal cancer patients diagnosed since May 1^st^, 2001. Data are provided from surgical departments, and include demographics, prognostic factors, and treatment data [31, 32].

Railway traffic noise was calculated as described above, for each person at time of death, and included in the study as a covariate only.

Finally, information on socioeconomic variables, e.g. education and income was calculated based on data from Statistics Denmark as the highest attained education and the income one year before cancer diagnosis. Similarly, Charlson Comorbidity Index [33] was calculated based on data from the Danish National Patient Registry [34] one year before cancer diagnosis.

We included lifestyle factors and socioeconomic characteristics as covariates in the models, whereas prognostic factors were included only in analyses of effect modification, as these are considered aspects of the underlying disease, appearing before the time-period between diagnosis and mortality, which is the focus of the present study.

### Statistical methods

Cox Proportional Hazards Models, estimating Mortality Rate Ratios (MRR), and 95% confidence intervals (CI) were used to investigate the association between residential traffic noise exposure and prognosis. Time since cancer diagnosis was used as the underlying time axis, and all analyses were stratified by age in 5-year intervals in order to allow for separate underlying hazards by age.

Subjects were followed from the date of colorectal cancer diagnosis, and until censoring due to death, loss to follow-up, or December 31^st^, 2013, whichever came first. We calculated the time-weighted average road traffic noise exposure over the entire follow-up period for each participant, taking into account all present and historical addresses in that period, as well as the individual length of follow-up time, so that a person who died was compared to a person who did not die, but had the same length of follow-up time. Furthermore, we modelled time-weighted average exposure over 1 year preceding death. This exposure measure was entered as a time-dependent variable into the statistical model.

The assumption of linearity of all continuous variables was evaluated visually, and by formal testing with linear spline models with boundaries forming three quartiles among deceased [35]. No deviation from linearity was observed when visually investigating the splines. The proportional hazards assumption of the Cox Models was tested by graphical inspection, which revealed no deviance from the assumption.

Mortality Rate Ratios were calculated as crude: Adjusted for age (by stratification), calendar year of diagnosis and sex, only (Model 1), and fully adjusted; i.e. as Model 1 and with additional adjustment for railway noise at diagnosis (0–20, >20–50, >50 dB), baseline smoking status (never, former, current, unknown), baseline smoking duration (linear, years), baseline alcohol intake (linear, g/day), baseline abstainers (yes, no), baseline red meat intake (linear, g/day), baseline recreational physical activity (yes, no), education 1 year before diagnosis (<7, 8–10, >10 years), and income 1 year before diagnosis (household income after taxation and interest, adjusted for number of persons in the household and divided into tertiles) (Model 2).

A mathematical description of the Cox models included can be found in supplementary material S1 Text.

Stratified analyses were conducted in order to investigate potential modification of the association between residential road traffic noise and mortality by age, sex, prognostic factors and comorbidity. This was evaluated by introducing interaction terms into the model, and were tested by the Wald test.

In analyses of colon and rectal cancer separately, the two outcomes were treated as competing causes of failure.

Sensitivity analysis were conducted to test the robustness of the main analysis by 1) excluding persons with missing information on included covariates (which were included in the main analyses as a separate missing-category), 2) by excluding those who died within the first two years of follow-up, in order to omit persons with so aggressive disease that the carcinogenic process may possibly be impervious to external circumstances, and 3) by ending follow-up at February 10^th^, 2012, which is the last date on which we had exposure information on the participants. In order to maximize statistical power, we followed up participants until December 31^st^, 2013, despite lack of updated exposure data in the main analyses.

All tests were based on the likelihood ratio test statistic. Two-sided 95% CI were calculated based on Wald’s test of the Cox regression parameter, i.e. on the log ratio scale. P-values < 0.05 were considered statistically significant. The procedure PHREG in SAS, version 9.3 was used for all statistical analyses (SAS Institute Inc., Cary, NC).

## Results

**Fig 1:**
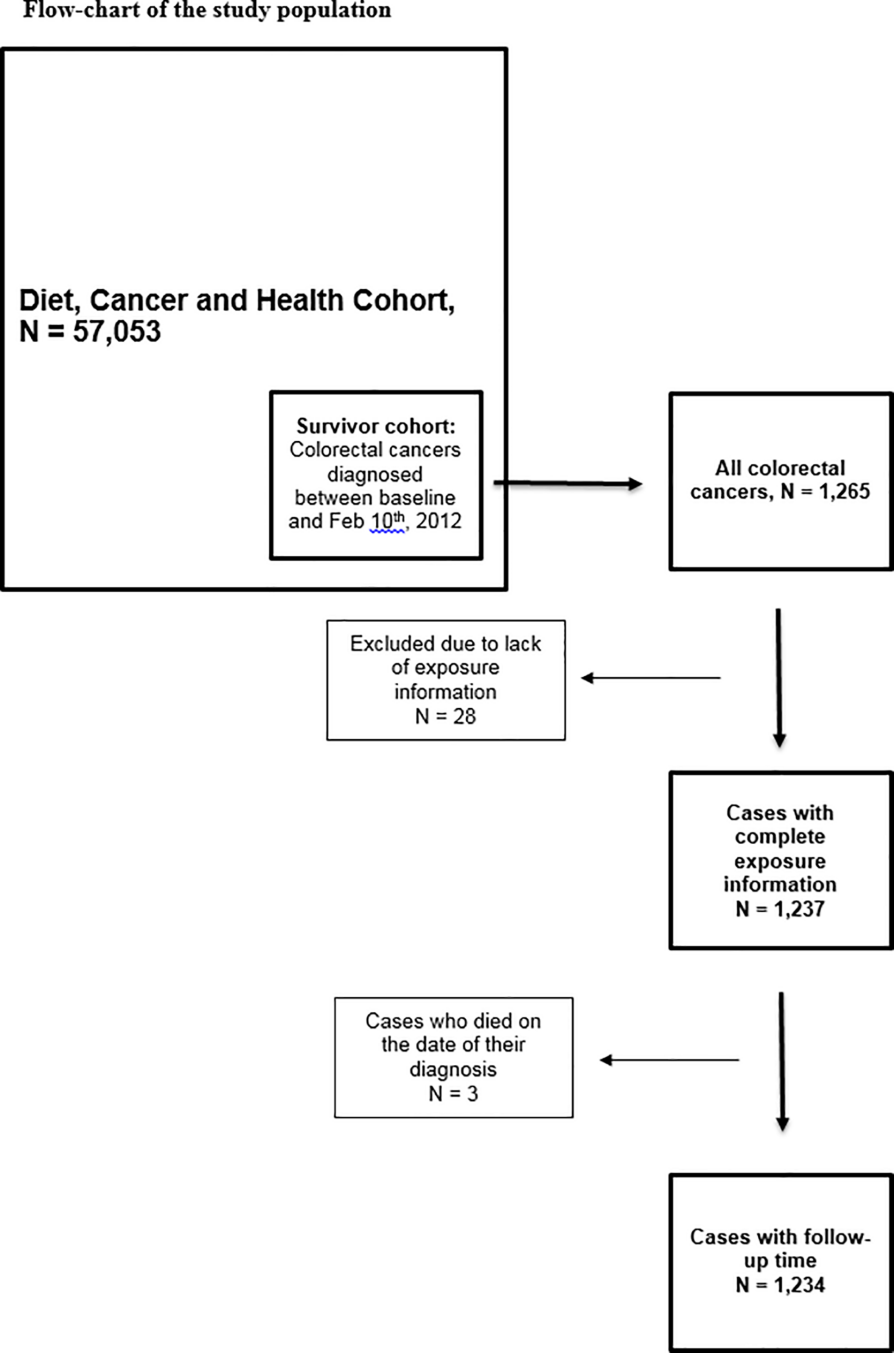
Flow-chart of the study population.

In the DCH-cohort, 1,265 persons were diagnosed with a first, primary colorectal cancer during follow-up. Of these, we excluded persons lacking information on exposure variables (n = 28), as well as cases who died on the day of their colorectal cancer diagnosis, and did thus not contribute any information to the models (n = 3). This left 1,234 persons (690 men, 544 women) for the present study (Fig 1).

During follow-up, 594 participants died, and of these, 75% died from colorectal cancer. Several of the potential confounding factors were unevenly distributed when comparing the entire cohort to those who died. Regardless of cause of death, those who died tended to have a shorter education and a lower income. They were more likely to be current smokers, and abstainers from alcohol, less likely to engage in recreational physical activity, and had a slightly higher intake of red meat. Those who died from colorectal cancer during follow-up were more likely to present with a higher malignancy grade and a larger extend of lymph node involvement at diagnosis, and the proportion of rectal cancers was somewhat smaller than among all colorectal cancer cases. Whereas those who died from any cause had a slightly higher median alcohol intake than the entire cohort, those who died from colorectal cancer had a slightly lower alcohol intake. The median follow-up time was 4 years for all colorectal cancer cases, but with very large variation. Those who died from colorectal cancer had a relatively short median survival time after diagnosis of only 1.5 years, which, however, also varied widely (**Table 1**).

**Table 1:** Characteristics of persons diagnosed with colorectal cancer in the Danish Diet, Cancer and Health cohort. All colorectal cancer caser patients, those who died during follow-up (All-cause mortality), and those who died from colorectal cancer (Colorectal cancer-specific mortality). Median and 5–95 percentile, unless otherwise stated.

	Colorectal cancer patients N = 1,234	All-cause mortality N = 594	Colorectal cancer-specific mortality N = 447
Follow-up time, years	4.0 (0.2–14.6)	1.9 (0.1–8.9)	1.5 (0.1–6.9)
Average road traffic noise over entire follow-up, dB	58.3 (50.0–70.3)	58.3 (50.1–69.8)	58.2 (49.8–69.9)
**Characteristics from time of diagnosis**			
Road traffic noise, dB	58.0 (49.6–70.7)	58.3 (49.8–70.0)	58.3 (49.8–69.9)
Railway noise, % exposed	21.1	18.7	19.9
Men, %	55.9	59.2	56.8
Age at diagnosis	67.0 (57.2–77.0)	66.4 (57.0–76.5)	66.3 (56.7–76.6)
Rectal cancer, %	35.3	34.7	32.9
Tumor stage, %			
*I*	11.4	3.9	1.8
*IIA + IIB*	22.0	10.2	7.8
*IIIA +IIIB + IIIC*	20.1	17.0	15.3
*IV*	16.7	30.5	36.0
*Missing*	29.8	38.4	38.0
Positive lymph nodes, %			
0	34.2	16.3	12.5
1–3	14.2	13.0	12.1
4–10	8.8	9.8	10.5
>10	4.1	6.2	7.6
*Missing*	38.7	54.7	57.3
**Variables 1 year before diagnosis**			
Education, %			
*< 7 years*	28.0	32.2	29.5
*8–10 years*	44.7	42.9	43.9
*>10 years*	26.2	23.7	25.3
*Missing*	1.2	1.2	1.3
Income, %^a^			
*1*^*st*^ *tertile*	19.0	23.1	21.0
*2*^*nd*^ *tertile*	32.2	32.0	32.2
*3*^*rd*^ *tertile*	48.8	45.0	46.8
Charlson Comorbidity Index > 0, n (%)	26.3	27.8	24.6
**Baseline lifestyle characteristics**			
Smoking status, %			
*Never*	29.7	25.7	27.4
*Former*	30.6	28.9	28.5
*Current*	39.6	45.4	44.3
Smoking duration, years	38 (22–48)	39 (22–49)	39 (22–49)
Alcohol, g/day^b^	15.2 (1.2–70.7)	15.4 (0.9–85.2)	14.7 (1.0–77.2)
*Abstainers*	33 (2.7)	24 (4.0)	18 (4.0)
Red meat intake, g/day	83.4 (33.7–169.6)	85.0 (35.9–178.3)	84.8 (33.0–179.0)
Recreational physical activity, %	48.0	42.2	45.0

^a^ household income after taxation and interest, adjusted for number of persons in the household and divided into tertiles.

^b^ Among those drinking alcohol.

**Table 2** presents the association between time-weighted average exposure 1 year preceding death, as well as over the entire follow-up period, and overall and colorectal cancer-specific mortality. Results are presented for all colorectal cancers, as well as for colon and rectal cancer separately. Regardless of exposure-window and outcome, we found no association between residential traffic noise exposure and colorectal cancer prognosis, e.g. the MRR for road traffic noise exposure over the entire follow-up period was 1.00 (0.88–1.13) per 10 dB.

**Table 2:** Crude and adjusted associations between residential road traffic noise exposure (L_den_) and overall and colorectal-cancer specific mortality, linear estimates per 10 dB.

	*All-cause mortality*			*Colorectal cancer specific mortality*		
	Deceased/ Total cases	*Model 1*^*a*^ MRR (95% CI)	*Model 2*^*b*^ MRR (95% CI)	Deceased/ Total cases	*Model 1*^*a*^ MRR (95% CI)	*Model 2*^*b*^ MRR (95% CI)
**All colorectal cancers**						
*Average L*_*den*_ *from diagnosis until censoring*	594/1,234	1.03 (0.92–1.17)	1.00 (0.88–1.13)	447/1,234	1.01 (0.88–1.16)	0.98 (0.85–1.13)
*Average L*_*den*_ *1 year before death*		1.02 (0.90–1.15)	0.99 (0.87–1.12)		1.00 (0.87–1.15)	0.98 (0.85–1.13)
**Colon cancers**						
*L*_*den*_ *from diagnosis until censoring*	388/799	1.02 (0.87–1.18)	0.98 (0.84–1.15)	300/799	1.01 (0.85–1.20)	0.97 (0.74–1.26)
*Average L*_*den*_ *1 year before death*		1.01 (0.87–1.17)	0.98 (0.84–1.16)		1.01 (0.85–1.20)	0.99 (0.83–1.18)
**Rectal cancers**						
*L*_*den*_ *from diagnosis until censoring*	206/435	1.03 (0.84–1.26)	1.03 (0.82–1.28)	147/435	0.95 (0.73–1.21)	0.97 (0.74–1.26)
*Average L*_*den*_ *1 year before death*		0.99 (0.81–1.22)	0.99 (0.79–1.23)		0.92 (0.72–1.18)	0.95 (0.72–1.24)

^a^ Adjusted for age (by stratification), sex, calendar-year (linear).

^b^ Adjusted for age (by stratification), sex, calendar-year (linear), train noise (0–20, >20–50, >50 dB),smoking status (never/former/current), smoking duration (linear, years), red meat intake (linear, g/day), alcohol intake (linear, g/day), abstainers (yes/no), recreational physical activity (yes/no), education (< 7/8-10/>10 years), income (household income after taxation and interest, adjusted for number of persons in the household and divided into tertiles).

When investigating interactions between residential traffic noise exposure and age, sex, prognostic factors, and Charlson score, we found a borderline significant interaction with malignancy grade and lymph node involvement, suggesting that those with less aggressive disease were more susceptible to traffic noise (MRR 1.29 (0.93–1.78) per 10 dB for tumor stage I-II; MRR 1.19 (0.86–1.63) per 10 dB for lymph node involvement). Charlson score, sex and age did not interact with traffic noise exposure (**Table 3**).

**Table 3:** Modification of the association between road traffic noise (per 10 dB) over entire follow-up period and risk of overall mortality by tumor characteristics, age, sex and Charlson score.

Covariates	N deceased/total	Model 2 MRR (95% CI)^a^	*P* interaction
**Tumor Stage**			
*I-II*	*84/411*	1.29 (0.93–1.78)	0.06
*III-IV*	*282/453*	0.90 (0.75–1.08)	
**Lymph node involvement**			
*Yes*	*172/333*	0.83 (0.63–1.05)	0.08
*No*	*97/421*	1.19 (0.86–1.63)	
**Sex**			
*Male*	351/688	0.99 (0.85–1.16)	0.92
*Female*	243/544	1.01 (0.83–1.22)	
**Age**			
*< median at baseline*	313/613	0.99 (0.84–1.17)	0.82
*≥ median at baseline*	281/619	1.04 (0.87–1.25)	
**Charlson Comorbidity Index**			
*0*	429/909	1.05 (0.90–1.22)	0.21
*>0*	165/323	0.89 (0.72–1.10)	

^a^ All analyses were adjusted for the basic covariates as in Table 2: age (by stratification), sex, calendar-year (linear), train noise (0–20, >20–50, >50 dB),smoking status (never/former/current), smoking duration (linear, years), red meat intake (linear, g/day), alcohol intake (linear, g/day), abstainers (yes/no), recreational physical activity (yes/no), education (< 7/8-10/>10 years), income (household income after taxation and interest, adjusted for number of persons in the household and divided into tertiles), however for the sub-analysis on effect modification by sex, sex was not included as a covariate.

We conducted sensitivity analyses to test the robustness of the results, and found only minor changes in the result of the main analysis when ending follow-up on February 10^th^, 2012, which was the last date of modelled noise, as well as when conducting a complete case analysis. Exclusion of persons who died within the first two years after diagnosis resulted in an MRR of 1.13 (0.94–1.35), per 10 dB over the entire follow-up period, in relation to overall mortality (**Table 4**).

**Table 4:** Sensitivity analyses of the association between road traffic noise (per 10 dB) over entire follow-up period, and risk of overall mortality.

	N deceased/total	Median follow-up time, years	Model 2 MRR (95% CI)^a^
Original estimate	595/1,235	4.0	0.99 (0.87–1.12)
Complete case analysis^b^	264/739	4.3	0.95 (0.79–1.15)
Exclusion of persons who die within two years of diagnosis	281/906	5.9	1.13 (0.94–1.35)
Ending follow-up on February 10^th^, 2012	527/1,235	3.0	0.99 (0.87–1.13)

^a^ Adjusted for age (by stratification), sex, calendar-year (linear), train noise (0–20, >20–50, >50 dB),smoking status (never/former/current), smoking duration (linear, years), red meat intake (linear, g/day), alcohol intake (linear, g/day), abstainers (yes/no), recreational physical activity (yes/no), education (< 7/8-10/>10 years), income (household income after taxation and interest, adjusted for number of persons in the household and divided into tertiles).

^b^ Excluding persons with missing information on tumor stage, lymph node involvement, and education.

## Discussion

The present study investigated the association between residential road traffic noise exposure and mortality in a cohort of Danish colorectal cancer patients. We found no association with overall or colorectal cancer-specific mortality. There was a borderline significant interaction between traffic noise and malignancy grade and lymph node involvement, suggesting a harmful effect of traffic noise among those with less clinically advanced disease. This analysis, was, however, hampered by small numbers.

The strengths of the present study include the virtually complete follow-up of the entire survivor-cohort through Danish registries on date [27] and cause of death [30], by means of the unique, Danish personal identification number. Furthermore, data on prognostic factors at time of diagnosis was available from the Danish Colorectal Cancer Group [31], and registry data on education and income was available from Danish health registries. These could possibly confound the association, as survival among colorectal cancer patients has been found related to socio-economic status [36, 37]. The Nordic Prediction Model [28], which we used to calculate noise, is well-known and the standard method for noise calculation in the Nordic countries, in studies on exposure and health. Access to complete address-history over follow-up allowed meticulous calculation of traffic noise exposure over different time-periods. Since no study has previously examined traffic noise exposure in relation to mortality in colorectal cancer patients, this was a hypothesis-generating study. We did not have an indication of which time-periods may be most relevant in relation to survival, and thus it was a major strength that we were able to investigate the association across different exposure-periods. We calculated also the association with a time-window of 5 years preceding death, but found similar results to 1 year preceding death, and this was thus not included in the tables.

The study was limited by a relatively low statistical power when examining colon and rectal cancer separately. The etiology of these two sub-sites is generally acknowledged to be diverse [38, 39], as is their treatment scheme, drug-response, and metastatic pattern [39]. Furthermore, a previous study in this cohort found a different effect of residential traffic noise on colon and rectal cancer incidence [40]. Taken together, this indicates that the effect of traffic noise in relation to prognosis might also differ. The lack of association for both sub-sites may be a true association, but could also be a result of too limited statistical power to detect an association. In order to maximize statistical power, we followed up participants until December 31^st^, 2013, despite only having exposure data until February 10^th^, 2012. Hence, we had a period of 22 moths with unknown exposure for participants who survived until the end of study. However, when conducting a sensitivity analysis, in which we ended follow-up on February 10^th^, 2012, we found virtually the same result as of the main analysis: MRR 0.99 (0.87–1.13) per 10 dB, for exposure from diagnosis to end of follow-up. We lacked information on colorectal cancer treatment in the present study, which is expected to have a larger effect on prognosis than traffic noise. However, given the highly standardized treatment of colorectal cancer patients in Denmark based on their tumor characteristics at time of diagnosis [32], we expect tumor characteristics to be plausible surrogate variables for this.

With regards to generalizability of the study findings, it should be noted that the participation rate in the original Diet, Cancer and Health study was only 35%, and a comparative study showed a considerable healthy-participant effect, with participants having a higher socio-economic position than non-participants [24]. Participants were selected from the two main urban areas of Denmark, Aarhus and Copenhagen, where we expect the traffic noise exposure to be relatively larger compared to the rest of Denmark. Furthermore, a study comparing mortality among participants and non-participants found that the cancer-specific mortality was significantly higher for non-participants; 35% for men and 52% for women, after adjustment for education and income [41].

To the best of our knowledge, this is the first study to examine the association between traffic noise exposure and survival among colorectal cancer patients, but a previous study in the same cohort suggested a harmful effect of residential traffic noise exposure on distal colon cancer incidence [40]. Due to limited power in the present study, we were, however, not able to examine sub-groups of colon cancer separately, but our results did not suggest an association with overall colon cancer. Another previous study in the Diet, Cancer and Health cohort recently investigated the association between traffic noise and survival among breast cancer patients, finding no overall association with all-cause or breast-cancer specific mortality. They did find an interaction with lymph node involvement suggesting a harmful effect among women with positive lymph nodes at diagnosis, but not women without [42]. The study agrees with the finding of no overall association between traffic noise and survival among cancer patients in the present study. However, the results of the interaction analyses go in pposite directions, as the present study suggest the strongest effect among those with less clinically advanced disease. These sub-findings may however be hampered by limited power, and could also be chance findings.

75% of the deaths recorded in the present cohort could be ascribed to colorectal cancer, and we did not see a difference in the association between traffic noise and all-cause or colorectal cancer-specific mortality, as the group of all-cause mortality was so heavily driven by colorectal cancer. We investigated both outcomes, as it is at present unknown through which biological pathway traffic noise may affect colorectal cancer prognosis: If it operates primarily through propulsion of carcinogenesis, we would primarily expect to see an association with colorectal cancer-specific mortality. However, consistent evidence has shown that traffic noise increases the risk of cardiovascular disease [43], and that cancer-treatment induces severe cardiovascular side-effects [44]; hence, if these factors interact, cancer patients could present an increased risk of cardiovascular mortality, rather than colorectal cancer-specific mortality. In the present study, we did not have the power to investigate cardiovascular mortality as a separate outcome, but would then have expected to see a stronger association with overall mortality than colorectal cancer-specific mortality. However, we did not find an association with any of the outcomes investigated.

In the adjusted models, we included a number of lifestyle-factors; smoking, alcohol, red meat intake, and physical activity, which are known to affect colorectal cancer prognosis [45, 46], but are also well-known risk-factors for colorectal cancer incidence [38]. Hence, these may be considered intermediate factors, and adjustment for them could be argued to remove part of their effect on eventual survival. However, when conducting analyses without adjustment for lifestyle, we found virtually identical results: MRR 1.00 (0.88–1.13), per 10 dB for overall mortality and 0.99 (0.86–1.14) per 10 dB for colorectal cancer-specific mortality, for exposure from diagnosis to end of follow-up.

We investigated effect modification by sex and age, as female sex and increasing age are associated with a poorer colorectal cancer prognosis [47], and as noise-effects on sleep may differ by sex [48], and sleep structure becomes more fragmented with increasing age [49], suggesting that age and sex could modify the effects of noise on colorectal cancer survival, through a pathway of sleep disruption. We further investigated effect modification by Charlson comorbidity index [33], as persons with multiple illnesses have a poorer colorectal cancer prognosis, and may be more susceptible to the effects of noise [4–6]. Finally, we investigated effect modification by prognostic variables; tumor stage and lymph node involvement, as these are of paramount importance for concurrent survival in colorectal cancer patients, and as we suspected that a potential harmful effect of traffic noise would be confined to patients with less advanced disease, in whom the carcinogenic process was still susceptible to external stimuli, such as traffic noise. We found no interaction with age, sex, or comorbidity, but we found borderline significant interactions with tumor stage and lymph node involvement, suggesting a harmful effect of traffic noise among those with less clinically advanced disease, only. This may be explained by studies showing that colorectal cancer patients with more advanced disease, measured by TNM-stage and lymph node involvement, have been found to display a more compromised circadian function, compared to those with less advanced disease [15, 18]. The circadian system affects a range of cellular processes which can affect cancer progression; including metabolism, cell cycle and DNA damage response [23, 50]. Hence, traffic noise may not be able to add further circadian load to this already severely disrupted group. The 5-years survival for patients with metastatic colorectal cancer is approximately 10% [51], suggesting a very limited preventive potential for this patient group. In contrast, among patients with less clinically advanced disease, and thus a smaller circadian disruption, the carcinogenic process may still be susceptible to external stimuli, so that those affected by traffic noise exposure develops a higher degree of circadian disruption, which has been found a strong marker for mortality in colorectal cancer patients [15, 16, 19–22]. However, the interaction analyses were generally hampered by small numbers in each cell, and the results are thus merely suggestive.

The majority of the patients in the present study presented with a colorectal cancer that had progressed beyond stage I already at time of diagnosis, and during a median follow-up time of 4 years, 48% of the participants died. The study had limited power, and hence we only investigated occurrence of mortality as a binary outcome. However, given the low survival-rate for colorectal cancer patients with advanced disease [51], future studies with a larger patient population could benefit from examining also residential traffic noise exposure in relation to more groups, defined by length of survival time.

We conducted a sensitivity analysis excluding those who died within the first two years of follow-up. Here, the estimate between traffic noise exposure and overall mortality was strengthened: MRR 1.13, 95% CI: 0.94–1.35. This corresponds well with the finding of an effect modification by prognostic factors, confining the detrimental consequences of residential traffic noise to those with less clinically advanced disease, with the estimate moving closer to the estimate for those with less clinically advance disease (Table 3), when they were excluded. However, the strengthening of the estimate could also be explained by an introduction of selection bias, and may thus not be a true association.

Information on tumor stage and lymph node involvement was missing for a relatively large number of participants; especially among those who died during follow-up. This is most likely due to the fact that there was no time to collect specimens from those who died shortly after being diagnosed. However, this should not affect the results of the main models of the present study, as we did not include clinicopathological features in these, since they were not considered covariates, but rather intermediary variables on the pathway from colorectal cancer to death. Instead, they were examined in interaction analyses, in which we only included participants with available data.

## Conclusion

In conclusion, the present study did not find an association between residential road traffic noise and survival in colorectal cancer patients. Interaction analyses did suggest that patients with less clinically advanced disease could be susceptible to harmful effects of traffic noise. However, these analyses were hampered by limited statistical power. As this is the first study of its kind, further studies are required to investigate the association before any firm conclusions can be drawn.

## Supporting information

S1 Text Supplementary material: Mathematical description of the Cox Models included. (DOCX)
