# Supplementary material for: Residential road traffic noise exposure and colorectal cancer survival – A Danish cohort study
Source: PLoS One. 2017 Oct 30;12(10):e0187161. doi: 10.1371/journal.pone.0187161 (PMC5662233; doi:10.1371/journal.pone.0187161)
Supplement: S1 Text — (DOCX) [file pone.0187161.s001.docx]

**S1 Text: Supplementary material: Mathematical description of the Cox Models included**

The mathematical formula for the Cox Model is:

h(t) = h_0_(t) * exp (b_1_x_1_ + b_2_x_2_ + _…_ + b_p_x_p_)

t represents the survival time and h_0_(t) denotes the hazard function as determined by the included covariates (x_1_, x_2_, …, x_p_)

The coefficients (b_1_, b_2_,...,b_p_) measure the impact (i.e., the effect size) of covariates

h_0_ is the baseline hazard

In the present study, we calculate the Cox Model for an association between road traffic noise as a time-varying variable, in relation to survival after colorectal cancer with two levels of adjustment:

**Model 1 – Crude.**

Adjusted only for age in 5-year groups (by stratification, in order to allow for different underlying baseline hazards), calendar year of diagnosis, and sex

As described by the below Cox Model:

h(t) = h_age5_(t) * exp ((Traffic noise(t), continuous) + (Calendar year, continuous) + (Sex, categorical))

**Model 2 – Adjusted.**

Adjusted as Model 1 i.e. for age (by stratification, in order to allow for different underlying baseline hazards), calendar year of diagnosis, and sex, and additionally for railway noise at diagnosis (0-20, >20-50, >50 dB), baseline smoking status (never, former, current, unknown), baseline smoking duration (linear, years), baseline alcohol intake (linear, g/day), baseline abstainers (yes, no), baseline red meat intake (linear, g/day), baseline recreational physical activity (yes, no), education 1 year before diagnosis (<7, 8-10, >10 years), and income 1 year before diagnosis (household income after taxation and interest, adjusted for number of persons in the household and divided into tertiles)

As described by the below Cox Model:

h(t) = h_age5_(t) * exp ((Traffic noise(t), continuous) + (Age, categorical) + (Calendar year, continuous) + (Sex, categorical) + (railway noise, categorical) + (smoking status, categorical) + (smoking duration, linear) + (alcohol intake, linear) + (abstainer, categorical) + (red meat intake, linear) + (recreational physical activity, categorical) + (education, categorical) + (income, categorical)

For both levels of adjustment, we calculated traffic noise at time t for two different time-periods, i.e. two different t:

Cumulated noise from t-1, t

Traffic noise (t) =

Cumulated noise from diagnosis to t
